# Extensive discohesive melanoma: additional challenges beyond the Breslow Index^☆^

**DOI:** 10.1016/j.abd.2025.501251

**Published:** 2025-12-29

**Authors:** Danielle Carvalho Quintella, Camila Sampaio Tomé Veloso-de-Araújo, Victor Faber, Ângela de Paiva Ansorge, Tullia Cuzzi

**Affiliations:** a Department of Pathology, Faculdade de Medicina, Universidade Federal de Rio de Janeiro, Rio de Janeiro, RJ, Brazil; b Pathology Service, Hospital Universitário Clementino Fraga Filho, Universidade Federal do Rio de Janeiro, Rio de Janeiro, RJ, Brazil; c Dermatology Service, Hospital Universitário Clementino Fraga Filho, Universidade Federal do Rio de Janeiro, Rio de Janeiro, RJ, Brazil

Dear Editor,

Discohesive,^1^ bullous,^2^, ^3^, ^4^ or acantholytic-like^5^ are terms that have been applied to the uncommon finding of variable morphological patterns of epidermal detachment^6^ associated with melanoma and referred to as the bullous variant.^2^ The main practical pathological problem associated with bullous melanoma regards the evaluation of the Breslow index. The authors argue about nomenclature and additional histopathological difficulties in a case qualified as an extensive discohesive melanoma.

A 64-year-old female patient, phototype II, was referred for evaluation of an asymptomatic lesion in the left scapular region. An asymmetrical multicolored brownish, blackened, grayish macular lesion with reddish areas, irregular edges, and approximately 6 centimeters was observed (Fig. 1). Dermoscopy showed atrophy, ulceration, irregular peripheral network, and globules.

**Figure 1:**
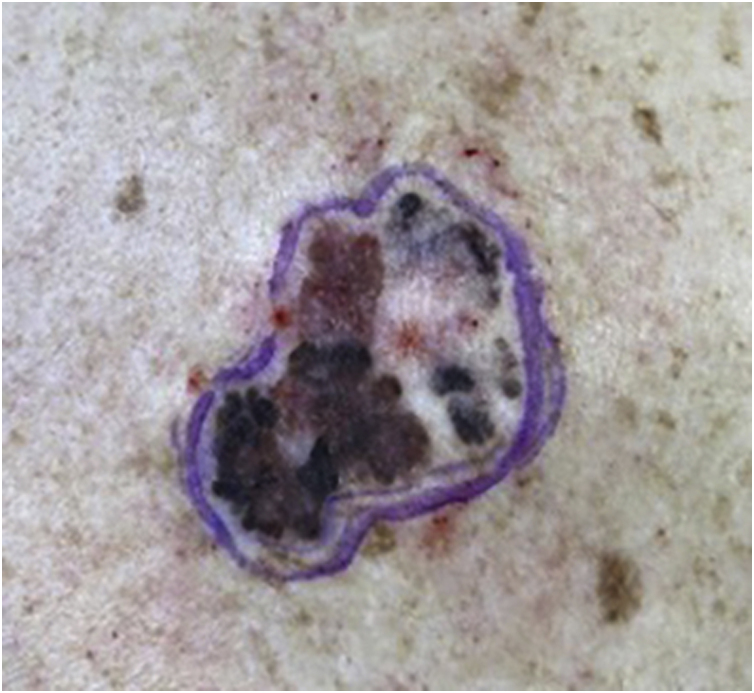
Asymmetrical multicolored brownish, blackened, grayish macular lesion with reddish areas and irregular edges.

The histopathological exam revealed atypical melanocyte proliferation along the dermal-epidermal junction. Most lesional segment was associated with clefts of varying length and width (Fig. 2), covered by intact or ruptured epidermis. Proliferated melanocytes were stellate with an expanded cytoplasm and enlarged, rounded and hyperchromatic nuclei seen in suprabasal position, along adnexal epithelium or located in the papillary dermis. Atypical melanocytes were variably present on the floor of the clefts (Fig. 3), on the roof, and/or floating inside the blister space, grouped or not. Other findings included two areas of true superficial ulceration (Fig. 3), an intense dermal lymphocytic inflammatory infiltrate with melanophages, focal fibroplasia, and mild solar elastosis. The diagnosis of extensive discohesive melanoma, superficial spreading type, with a Breslow index of 1 mm, ulceration, and incomplete regression was considered. Immunostaining with Melan-A was performed to better assess the histopathological findings (Fig. 4).

**Figure 2:**
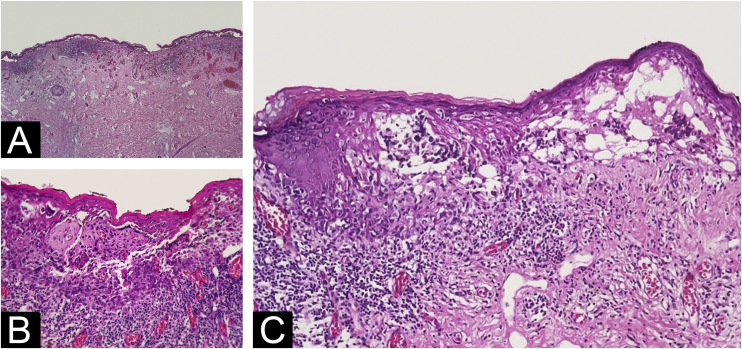
Varying degrees of dermal-epidermal detachment along almost de whole lesion (A – Hematoxylin & eosin, ×4); intact epidermis and grouped malignant melanocytes in the bottom, where superficial dermal infiltration is present (B ‒ Hematoxylin & eosin, ×10); intraepidermal (on the left) and subepidermal (on the right) multilocular cleft with pagetoid dissemination of isolated cells that reached the granulosa layer (C ‒ Hematoxylin & eosin, ×10).

**Figure 3:**
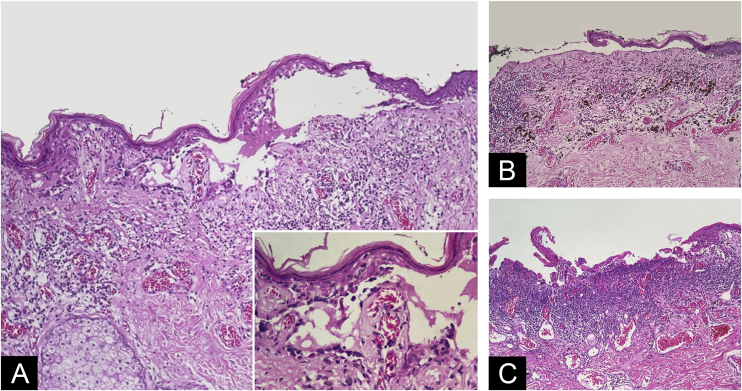
Blister with melanocytes present on the floor of the cleft, sometimes carpeting the dermis (A ‒ Hematoxylin & eosin, ×10; Insert: detail of carpeting melanocytes ‒ Hematoxylin & eosin, ×40); subepidermal ruptured bulla devoid of melanocytes at the bottom above dermal regressive changes (B ‒ Hematoxylin & eosin, ×10); necrotic basophilic material covering papillary dermis together with juxtaposed inflammatory infiltrate. Some discohesive melanocytes can be seen in the lateral border of necrotic tissue (C ‒ Hematoxylin & eosin, ×10).

**Figure 4:**
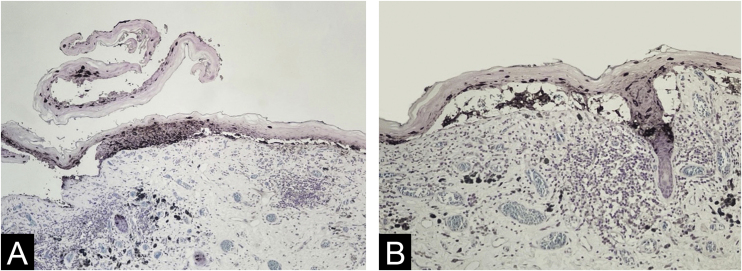
Melanocytes occupying almost the whole epidermal thickness at the lateral border of a ruptured blister devoid of melanocytes seen only on its roof (on the left) and carpeting melanocytes in the bottom of a narrow cleft (on the right) (A ‒ Melan-A immunostaining, Giemsa counter-staining, ×10); melanocytes floating in cleft space and among keratinocytes at the roof. Notice epithelium-stroma detachment in follicular infundibula. (B ‒ Melan-A immunostaining, Giemsa counter-staining, ×10).

Discohesion among proliferating melanocytes and clefts are clues to the diagnosis of melanoma as compared to benign melanocytic proliferation.^6^ Loss of adhesion of the neoplastic cells is attributed to the downregulation of receptors like E-cadherin and altered expression of integrin family molecules.^7^ Meanwhile, melanoma with large, wide, or extensive clefts, with or without clinical expression, can be qualified as discohesive melanoma.^1^, ^3^, ^4^, ^5^ In the present case, despite no clinical observation of bulla, varying degrees of epidermal detachment composed most of the lesion length. Discohesive, isolated, round malignant cells could be referred to as acantholytic-like, but the finding was not so prominent as previously reported,^5^ and the designation “bullous” was avoided. In addition, a Hailey-Hailey disease-like pattern, considering attached melanocytes floating in the clefts, was focal.^1^, ^4^

Melan-A immunostaining was useful in the visualization of melanocytes in the roof of ruptured clefts, particularly when the denuded dermis lacked both melanocytes and keratinocytes, and the picture can mimic a primary blister disorder.

Extensive epidermal detachment, distorting epidermal architecture, made it difficult to histologically classify the lesion. It was considered a superficial spreading melanoma due to a high-level pagetoid spread observed in the short segments where the epidermis was still intact and attached to the dermis, and the absence of dermal changes that characterize significant chronic sun damage.

The Breslow index is the main practical problem posed by melanomas with clefts. While some have included the bulla space in the Breslow index,^5^, ^8^ others suggest excluding the area.^4^ The inclusion of the cleavage area resulted in a thickness of 1 mm, while its exclusion resulted in 0.9 mm, thus not affecting the pathological staging (pT1b/AJCC, 2018) in this case. The question is not addressed in recent guideline publications^9^, ^10^ and the Breslow index measured in the context of extensive discohesive melanoma will imply a probable over- or under-measurement to be declared in the pathological report.

Additional histopathological challenges account for the evaluation of ulceration. Detached segments with a ruptured roof, with melanocytes present only in their handedness or in the overlying epidermis, leaving behind a smooth papillary dermis devoid of cells and juxtaposed inflammation, were not considered true ulceration. On the other hand, consistent with dermoscopy findings, two areas were interpreted as actual superficial ulceration based on projections of degenerated papillary dermis or necrotic aspect of the floor along with a greater accumulation of inflammatory cells, including neutrophils, and fibrin.^10^

Melanoma clefts and blisters may create exceptional difficulties in the evaluation of several histopathological parameters that are not restricted to the measurement of Breslow's index but also concern the classification of the *in-situ* component of the lesion and the indication of ulceration, all aspects linked to clinical evolution, prognosis, and staging.

## ORCID ID

Camila Sampaio Tomé Veloso-de-Araújo: 0009-0001-2348-4269

Victor Faber: 0009-0008-7088-5832

Ângela de Paiva Ansorge: 0009-0004-9060-0962

Tullia Cuzzi: 0000-0002-3331-5290

## Research data availability

Does not apply.

## Financial support

This research did not receive any specific grant from funding agencies in the public, comercial, or not-for-profit sectors.

## Authors' contributions

Danielle Carvalho Quintella: Approval of the final version of the manuscript; critical literature review; data collection, analysis and interpretation; effective participation in research orientation; manuscript critical review; preparation and writing of the manuscript.

Camila Sampaio Tomé Veloso-de-Araújo: Approval of the final version of the manuscript; critical literature review; data collection, analysis and interpretation; manuscript critical review; preparation of the manuscript.

Victor Faber: Approval of the final version of the manuscript; critical literature review; data collection, analysis and interpretation; manuscript critical review; preparation of the manuscript.

Ângela de Paiva Ansorge: Approval of the final version of the manuscript; critical literature review; data collection, analysis and interpretation; manuscript critical review; preparation and writing of the manuscript.

Tullia Cuzzi: Approval of the final version of the manuscript; critical literature review; data collection, analysis and interpretation; effective participation in research orientation; manuscript critical review; preparation and writing of the manuscript; study conception and planning.

## Conflicts of interest

None declared.
